# Association of Receipt of Opioid Use Disorder–Related Telehealth Services and Medications for Opioid Use Disorder With Fatal Drug Overdoses Among Medicare Beneficiaries Before and During the COVID-19 Pandemic

**DOI:** 10.1001/jamapsychiatry.2023.0310

**Published:** 2023-03-29

**Authors:** Christopher M. Jones, Carla Shoff, Carlos Blanco, Jan L. Losby, Shari M. Ling, Wilson M. Compton

**Affiliations:** 1 National Center for Injury Prevention and Control, Centers for Disease Control and Prevention, Atlanta, Georgia; 2 Office of the Administrator, Centers for Medicare & Medicaid Services, Baltimore, Maryland; 3 National Institute on Drug Abuse, National Institutes of Health, Bethesda, Maryland; 4 Centers for Clinical Standards and Quality, Centers for Medicare & Medicaid Services, Baltimore, Maryland

## Abstract

**Importance:**

Federal emergency authorities were invoked during the COVID-19 pandemic to expand clinical telehealth for opioid use disorder (OUD).

**Objective:**

To examine the association of the receipt of telehealth services and medications for OUD (MOUD) with fatal drug overdoses before and during the pandemic.

**Design, Setting, and Participants:**

This cohort study used exploratory longitudinal data from 2 cohorts (prepandemic cohort: September 1, 2018, to February 29, 2020; pandemic cohort: September 1, 2019, to February 28, 2021) of Medicare Fee-for-Service beneficiaries aged 18 years or older initiating an episode of OUD-related care using Medicare Fee-for-Service data from the Centers for Medicare & Medicaid Services and National Death Index data from the Centers for Disease Control and Prevention. Data analysis was performed from September 19 to October 17, 2022.

**Exposures:**

Prepandemic vs pandemic cohort demographic, medical, substance use, and psychiatric characteristics.

**Main Outcomes and Measures:**

Receipt of OUD-related telehealth services, receipt of MOUD, and fatal drug overdose.

**Results:**

The prepandemic cohort comprised 105 162 beneficiaries (58.1% female; 67.6% aged 45-74 years). The pandemic cohort comprised 70 479 beneficiaries (57.1% female; 66.3% aged 45-74 years). The rate of all-cause mortality was higher in the pandemic cohort (99.9 per 1000 beneficiaries; 7041 deaths) than in the prepandemic cohort (76.8 per 1000; 8076 deaths) (*P* < .001). The rate of fatal drug overdoses was higher in the pandemic cohort (5.1 per 1000 beneficiaries; n = 358) than in the prepandemic cohort (3.7 per 1000; n = 391) (*P* < .001). The percentage of deaths due to a fatal drug overdose was similar in the prepandemic (4.8%) and pandemic (5.1%) cohorts (*P* = .49). In multivariable analysis of the pandemic cohort, receipt of OUD-related telehealth was associated with a significantly lower adjusted odds ratio (aOR) for fatal drug overdose (aOR, 0.67; 95% CI, 0.48-0.92) as was receipt of MOUD from opioid treatment programs (aOR, 0.41; 95% CI, 0.25-0.68) and receipt of buprenorphine in office-based settings (aOR, 0.62; 95% CI, 0.43-0.91) compared with those not receiving MOUD; receipt of extended-release naltrexone in office-based settings was not associated with lower odds for fatal drug overdose (aOR, 1.16; 95% CI, 0.41-3.26).

**Conclusions and Relevance:**

This cohort study found that, among Medicare beneficiaries initiating OUD-related care during the COVID-19 pandemic, receipt of OUD-related telehealth services was associated with reduced risk for fatal drug overdose, as was receipt of MOUD from opioid treatment programs and receipt of buprenorphine in office-based settings. Strategies to expand provision of MOUD, increase retention in care, and address co-occurring physical and behavioral health conditions are needed.

## Key Points

**Question:**

Were new federal authorities to expand telehealth use for substance use disorder treatment and to facilitate provision of medications for opioid use disorder (MOUD) during the COVID-19 pandemic associated with a lower risk for fatal drug overdose among Medicare beneficiaries initiating a new episode of opioid use disorder (OUD)–related care?

**Findings:**

In this cohort study of a prepandemic cohort comprising 105 162 beneficiaries and a pandemic cohort comprising 70 479 beneficiaries, receipt of OUD-related telehealth services and receipt of MOUD during the pandemic were associated with reduced odds for fatal drug overdose.

**Meaning:**

This study found that emergency authorized telehealth expansion and MOUD provision during the COVID-19 pandemic were associated with lower odds of fatal drug overdose, demonstrating the potential benefits of continuing these services.

## Teaser

This cohort study examines the association of the receipt of telehealth services and medications for opioid use disorder (OUD) with fatal drug overdoses before and during the pandemic.

## Introduction

During the COVID-19 pandemic, federal actions were taken to facilitate access to care for individuals with opioid use disorder (OUD), including expanded telehealth, remote prescribing of buprenorphine, and expanded take-home doses of methadone from opioid treatment programs (OTPs).^1,2,3,4,5,6^

We previously used a longitudinal 2-cohort study design with Medicare Fee-for-Service (FFS) data from September 2018 to February 2021 to compare the use of telehealth services among Medicare beneficiaries initiating OUD-related care before and during the pandemic.^7^ Use of OUD-related telehealth services during the pandemic was associated with improved retention in use of medications for opioid use disorder (MOUD) and reduced risk for medically treated overdose.^7^ Examining fatal drug overdoses was not possible due to lack of linked Medicare and overdose death data at that time. With the recent availability of linked data through 2021, this follow-up study uses the same 2-cohort longitudinal design to examine the association of receipt of OUD-related telehealth services and MOUD with drug overdose deaths before and during the COVID-19 pandemic.

## Methods

Multiple Centers for Medicare & Medicaid Services (CMS) data sources^8,9^ were used (eTable in Supplement 1). Drug overdose death data from the Centers for Disease Control and Prevention (CDC) National Death Index were linked to Medicare Beneficiary Summary Files. The full details of the cohort design have been provided elsewhere.^7^ In brief, we constructed 2 mutually exclusive cohorts of Medicare FFS beneficiaries aged 18 years or older with OUD, both before and during the pandemic, consisting of individuals starting new (index) episodes of OUD-related care. Individuals already receiving OUD-related services identified by *International Classification of Diseases, Ninth Revision, Clinical Modification* (*ICD-10-CM*) diagnosis codes for OUD during the 6-month cohort preindex period were excluded. The 6-month preindex period (prepandemic cohort: September 2018 to February 2019; pandemic cohort: September 2019 to February 2020) was followed by a 6-month index period (prepandemic cohort: March 2019 to August 2019; pandemic cohort: March 2020 to August 2020) when beneficiaries were enrolled in each cohort, then a 6-month cohort follow-up period (prepandemic cohort: September 2019 to February 2020; pandemic cohort: September 2020 to February 2021)^10^ (eFigure in Supplement 1). This cohort study was covered by the Common Rule exemption, 45 CFR 46.104(d)(4)(iv), and did not require institutional review board review (ie, the data were deidentified). This study followed the Strengthening the Reporting of Observational Studies in Epidemiology (STROBE) reporting guideline.

### Outcomes

The main outcomes were (1) receipt of OUD-related telehealth services; (2) receipt of MOUD (MOUD from OTPs, buprenorphine in office-based settings, extended-release (ER) naltrexone in office-based settings); and (3) fatal drug overdose. Receipt of OUD-related telehealth services was defined as a Medicare Part A or B service claim with OUD diagnosis and telehealth service code, defined by either Place of Service code 02 and/or a combination of Healthcare Common Procedure Coding System (HCPCS) modifier codes and HCPCS or Current Procedural Terminology codes on the claim. Receipt of MOUD from OTPs was defined as having a Part B claim with HCPCS codes corresponding to receipt of MOUD. Receipt of buprenorphine or ER naltrexone in office-based settings was defined as having a Part D pharmacy claim with a National Drug Code for buprenorphine products approved for OUD treatment or for ER naltrexone. Drug overdose deaths were identified with *International Statistical Classification of Diseases and Related Health Problems, Tenth Revision* codes underlying cause of death (X40-44, X60-64, X85, and Y10-14) and occurring after the beneficiary entered the cohort (eTable in Supplement 1).

### Statistical Analysis

Baseline demographic and clinical characteristics were examined by cohort and reported as frequencies and percentages. The number and rate of deaths per 1000 beneficiaries from all causes and the number of deaths due to drug overdose and the percentage of all deaths due to drug overdose were examined by cohort. To assess differences between cohorts, we used the χ^2^ test for proportions and percentages.

For the pandemic cohort, a multilevel logistic regression model examined associations between fatal drug overdose during the study period and receipt of OUD-related telehealth services and MOUD. The model was adjusted for baseline characteristics, percentage of days during study period patients received any OUD-related services, and percentage of days patients received any non-OUD–related services; beneficiary state of residence was included as a level 2 random intercepts parameter to adjust for similarity of beneficiaries residing in the same state. Results are presented as adjusted odds ratios (aORs) and corresponding 95% CIs. Multicollinearity was not identified in final models. Statistical significance was set at *P* <  .05, and all *P* values were 2-tailed. Analyses were conducted with SAS Enterprise Guide, version 7.1 (SAS Institute Inc) and Stata, version 17.0 (StataCorp).

## Results

The sociodemographic and clinical characteristics of the prepandemic (n = 105 162) and pandemic (n = 70 479) cohort beneficiaries are presented in Table 1. A larger percentage of the beneficiaries in the pandemic cohort received OUD-related telehealth services than beneficiaries in the prepandemic cohort (13 809 beneficiaries [19.6%] vs 590 beneficiaries [0.6%]; *P* < .001). Receipt of MOUD was higher in the pandemic cohort than in the prepandemic cohort (8826 beneficiaries [12.6%] vs 11 304 beneficiaries [10.8%]; *P* < .001).

**Table 1.** Baseline Demographic and Clinical Characteristics of Medicare Fee-for-Service Beneficiaries With Opioid Use Disorder, by Cohort

Characteristic	Beneficiaries, No. (%)^a^		*P* value^b^
	Pre–COVID-19 pandemic cohort	COVID-19 pandemic cohort	
Cohort size	105 162 (59.9)	70 479 (40.1)	NA
Sex			
Female	61 108 (58.1)	40 227 (57.1)	<.001
Male	44 054 (41.9)	30 252 (42.9)	
Age group, y			
18-44	11 762 (11.2)	7774 (11.0)	<.001
45-64	37 487 (35.6)	22 966 (32.6)	
65-74	33 612 (32.0)	23 788 (33.8)	
≥75	22 301 (21.2)	15 951 (22.6)	
Race and ethnicity			
Hispanic	6443 (6.2)	4417 (6.4)	<.001
Non-Hispanic African American	12 382 (11.9)	7863 (11.3)	
Non-Hispanic White	82 765 (79.5)	55 464 (79.8)	
Non-Hispanic other^c^	2478 (2.4)	1807 (2.6)	
US Census region			
Northeast	18 479 (17.6)	11 061 (15.7)	<.001
Midwest	19 826 (18.9)	12 724 (18.1)	
South	41 642 (39.6)	29 089 (41.3)	
West	25 196 (24.0)	17 594 (25.0)	
County urban-rural status			
Metropolitan	81 376 (77.4)	55 221 (78.4)	<.001
Micropolitan	21 134 (20.1)	13 594 (19.3)	
Rural	2644 (2.51)	1655 (2.4)	
Dual status			
Medicare only	47 834 (45.5)	33 761 (47.9)	<.001
Medicare and Medicaid	57 328 (54.5)	36 718 (52.1)	
Eligibility			
Aged ≥65 y	55 140 (52.4)	39 004 (55.3)	<.001
Disability	47 676 (45.3)	29 497 (41.8)	
ESRD	2346 (2.2)	1978 (2.8)	
Other substance use disorder diagnosis			
Alcohol	12 195 (11.6)	8204 (11.6)	.78
Tobacco	38 707 (36.8)	24 156 (34.3)	<.001
Cannabis	7809 (7.4)	5228 (7.4)	.95
Cocaine	4720 (4.5)	2903 (4.1)	<.001
Stimulant	5025 (4.8)	3620 (5.1)	<.001
Sedative or hypnotic	6628 (6.3)	4679 (6.6)	.01
Other psychoactive substance	12 339 (11.7)	7580 (10.8)	<.001
≥2 Substances	20 018 (19.0)	12 927 (18.3)	<.001
Mental health diagnosis			
Anxiety	58 633 (55.8)	39 130 (55.5)	.33
Bipolar disorder	17 442 (16.6)	11 727 (16.6)	.77
Major depression	58 238 (55.4)	38 290 (54.3)	<.001
Personality disorder	6663 (6.3)	4260 (6.0)	.01
Attention-deficit/hyperactivity disorder	5317 (5.1)	3541 (5.0)	.76
Posttraumatic stress disorder	8891 (8.4)	5951 (8.4)	.94
Schizophrenia or other psychotic disorder	9843 (9.4)	6928 (9.8)	.001
≥2 Mental health diagnoses	49 991 (47.5)	33 149 (47.0)	.04
Other chronic medical conditions			
Cancer	11 334 (10.8)	7913 (11.2)	.003
Diabetes	38 042 (36.2)	25 523 (36.2)	.87
Cardiovascular and other circulatory	81 056 (77.1)	54 130 (76.8)	.18
Chronic respiratory disease	40 322 (38.3)	25 742 (36.5)	<.001
Viral hepatitis	9085 (8.6)	5447 (7.7)	<.001
HIV	1434 (1.4)	963 (1.4)	.96
Obesity	38 722 (36.8)	25 511 (36.2)	.008
Liver disease cirrhosis and other liver conditions^d^	14 111 (13.4)	9698 (13.8)	.04
Acute or chronic pain	93 548 (89.0)	61 861 (87.8)	<.001

Abbreviations: ESRD, end-stage renal disease; NA, not applicable.

^a^ Seventy-five beneficiaries were excluded from the study due to dying on the same day as an opioid use disorder index date (40 in the prepandemic cohort and 35 in the pandemic cohort).

^b^ To assess differences between cohorts, we used the χ^2^ test for proportions and percentages.

^c^ Included American Indian or Alaska Native, Asian or Pacific Islander, and other race.

^d^ Excluding hepatitis.

The all-cause mortality rate was higher in the pandemic cohort than the prepandemic cohort (99.9 [95% CI, 97.8-102.2] vs 76.8 [95% CI, 75.2-78.5] deaths per 1000 beneficiaries; *P* < .001) (Table 2). The rate of drug overdose deaths was also higher in the pandemic cohort (5.1 [95% CI, 4.6-5.6] deaths per 1000 beneficiaries) than in the prepandemic cohort (3.7 [95% CI, 3.4-4.1] deaths per 1000 beneficiaries) cohort (*P* < .001). The percentage of deaths due to drug overdoses did not differ between the cohorts (4.8% (95% CI, 4.4%-5.3%) in the prepandemic cohort and 5.1% (95% CI, 4.6%-5.6%) in the pandemic cohort; *P* = .49).

**Table 2.** All-Cause Mortality and Drug Overdose Mortality Among Medicare Fee-for-Service Beneficiaries With Opioid Use Disorder^a^

Type of mortality	Pre–COVID-19 pandemic cohort (n = 105 162)	COVID-19 pandemic cohort (n = 70 479)	*P* value^b^
All-cause mortality			
No. of deaths	8076	7041	
Rate of death per 1000 beneficiaries (95% CI)	76.8 (75.2-78.5)	99.9 (97.8-102.2)	<.001
Drug overdose mortality			
No. of deaths	391	358	
Rate of death per 1000 beneficiaries (95% CI)	3.7 (3.4-4.1)	5.1 (4.6-5.6)	<.001
% of All deaths due to drug overdose (95% CI)	4.8 (4.4-5.3)	5.1 (4.6-5.6)	.49

^a^ Seventy-five beneficiaries were excluded from the study due to dying on the same day as an opioid use disorder index date (40 in the prepandemic cohort and 35 in the pandemic cohort).

^b^ To assess differences between cohorts, we used the χ^2^ test for proportions and percentages.

In the pandemic cohort, a lower adjusted odds of fatal drug overdose was found among beneficiaries receiving OUD-related telehealth services (aOR, 0.67; 95% CI, 0.48-0.92) and those receiving MOUD from OTPs (aOR, 0.41; 95% CI, 0.25-0.68) and buprenorphine in office-based settings (aOR, 0.62; 95% CI, 0.43-0.91) compared with receiving no MOUD (Table 3). Certain demographic and clinical characteristics were also associated with fatal drug overdoses.

**Table 3.** Characteristics Associated With Fatal Drug Overdose During Study Period Among Beneficiaries With Opioid Use Disorder in the Pandemic Cohort^a^

Characteristic	Beneficiaries, No. (%)	aOR (95% CI)^b^
Receipt of OUD-related telehealth service	13 809 (19.6)	0.67 (0.48-0.92)^c^
Receipt of MOUD during study period		
No MOUD	61 626 (87.5)	1 [Reference]
MOUD from OTPs	2774 (3.9)	0.41 (0.25-0.68)^c^
ER naltrexone in office-based settings	170 (0.2)	1.16 (0.41-3.26)
Buprenorphine in office-based settings	5882 (8.4)	0.62 (0.43-0.91)^c^
Baseline sex		
Male	30 233 (42.9)	1 [Reference]
Female	40 219 (57.1)	0.54 (0.43-0.68)^c^
Baseline age group, y		
18-44	7760 (11.0)	1 [Reference]
45-64	22 953 (32.6)	0.84 (0.64-1.11)
65-74	23 788 (33.8)	0.37 (0.25-0.55)^c^
75 or older	15 951 (22.6)	0.14 (0.07-0.28)^c^
Baseline race and ethnicity		
Hispanic	4417 (6.4)	0.78 (0.49-1.23)
Non-Hispanic African American	7862 (11.3)	0.88 (0.62-1.23)
Non-Hispanic White	55 440 (79.7)	1 [Reference]
Non-Hispanic other^d^	1806 (2.6)	0.90 (0.45-1.80)
Baseline US Census region		
Northeast	11 056 (15.7)	1 [Reference]
Midwest	12 716 (18.1)	0.84 (0.55-1.28)
South	29 078 (41.3)	0.72 (0.49-1.06)
West	17 591 (25.0)	0.73 (0.46-1.15)
Baseline county urban-rural status		
Metropolitan	55 200 (78.4)	1 [Reference]
Micropolitan	13 589 (19.3)	0.75 (0.55-1.02)
Rural	1654 (2.4)	0.78 (0.34-1.79)
Baseline dual status		
Medicare only	33 760 (47.9)	1 [Reference]
Medicare and Medicaid	36 692 (52.1)	1.30 (0.97-1.74)
Time spent in nursing home from baseline to end of study		
No time	61 707 (87.6)	1 [Reference]
Quantile 1	2126 (3.0)	0.54 (0.27-1.10)
Quantile 2	2053 (2.9)	0.31 (0.10-0.97)^c^
Quantile 3 or 4	4566 (6.5)	0.24 (0.10-0.55)^c^
Baseline other substance use disorder diagnosis^e^		
Alcohol	8191 (11.6)	1.01 (0.77-1.32)
Tobacco	24 133 (34.2)	1.65 (1.27-2.13)^c^
Cannabis	5218 (7.4)	0.73 (0.54-0.99)^c^
Cocaine	2894 (4.1)	1.70 (1.25-2.32)^c^
Stimulant	3611 (5.1)	1.00 (0.72-1.38)
Sedative or hypnotic	4674 (6.6)	1.68 (1.23-2.29)^c^
Other psychoactive substance	7567 (10.7)	2.48 (1.90-3.23)^c^
Baseline mental health diagnosis^e^		
Anxiety	39 109 (55.5)	1.30 (0.99-1.71)
Bipolar disorder	11 717 (16.6)	1.20 (0.91-1.57)
Major depression	38 267 (54.3)	1.03 (0.80-1.32)
Personality disorder	4258 (6.0)	1.08 (0.76-1.54)
Attention-deficit/hyperactivity disorder	3539 (5.0)	0.81 (0.56-1.18)
Posttraumatic stress disorder	5944 (8.4)	0.90 (0.65-1.24)
Schizophrenia or other psychotic disorder	6923 (9.8)	0.81 (0.59-1.10)
Baseline other chronic medical conditions^e^		
Cancer	7913 (11.2)	0.47 (0.28-0.80)^c^
Diabetes	25 521 (36.2)	1.03 (0.80-1.33)
Cardiovascular and other circulatory	54 119 (76.8)	0.81 (0.62-1.05)
Chronic respiratory disease	25 733 (36.5)	1.14 (0.90-1.45)
Viral hepatitis	5441 (7.7)	1.52 (1.15-2.00)^c^
HIV	963 (1.4)	0.65 (0.33-1.31)
Obesity	25 506 (36.2)	0.71 (0.55-0.91)^c^
Liver disease cirrhosis and other liver conditions^f^	9696 (13.8)	1.22 (0.92-1.62)
Acute or chronic pain	61 874 (87.8)	0.63 (0.48-0.82)^c^
% of Days receiving care from baseline to end of study or death		
OUD-related care	NA	1.04 (1.03-1.05)^c^
Non-OUD–related care	NA	1.01 (1.01-1.02)^c^

Abbreviations: aOR, adjusted odds ratio; ER, extended release; MOUD, medications for opioid use disorder; NA, not applicable; OTP, opioid treatment program; OUD, opioid use disorder.

^a^ To enable mutually exclusive groups for the regression analysis, 27 beneficiaries (0.30% of beneficiaries receiving MOUD) who received both buprenorphine and ER naltrexone from pharmacies during the study period were excluded from the analysis. Also, 75 beneficiaries were excluded from the study due to dying on the same day as an opioid use disorder index date (40 in the prepandemic cohort and 35 in the pandemic cohort).

^b^ Models adjusted for all variables.

^c^ *P* ≤ .05.

^d^ Included American Indian or Alaska Native, Asian or Pacific Islander, and other race.

^e^ Reference group does not have a condition.

^f^ Excluding hepatitis.

## Discussion

Medicare beneficiaries starting a new episode of OUD-related care during the pandemic who received OUD-related telehealth services had a 33% lower adjusted odds of a fatal drug overdose, even after accounting for OUD and non-OUD care engagement and receipt of MOUD. This is consistent with our previous study that found OUD-related telehealth services were associated with reduced risk for nonfatal overdose.^7^ We also found that receipt of MOUD from OTPs and receipt of buprenorphine in office-based settings were associated with 59% and 38% lower adjusted odds of fatal drug overdose, respectively. In contrast, consistent with prior research,^11,12^ receipt of ER naltrexone was not associated with a lower risk for overdose death, nor with a lower risk for nonfatal overdose in our prior study.^7^

All-cause mortality and drug overdose deaths were higher in the pandemic cohort compared with the prepandemic cohort. This increase in overdose deaths among the pandemic cohort is consistent with national trends showing a 31% increase in overdose deaths between 2019 and 2020.^13^

Despite encouraging findings associated with telehealth and MOUD, only 1 in 5 Medicare beneficiaries in our pandemic cohort received OUD-related telehealth services, and only 1 in 8 received MOUD, underscoring the need for continued expansion of these potentially life-saving interventions across clinical settings.^14,15^ In addition, our study highlights several findings for future research. For example, exploring why tobacco use disorders were associated with an increased risk, cannabis use disorders with a decreased risk, and alcohol use disorders not associated with a risk for fatal overdose is warranted. Similarly, cancer, obesity, and pain diagnoses were all associated with a lower risk for fatal overdose.

### Limitations

Our study contains Medicare FFS beneficiaries that sought and received care before and during the pandemic; the findings might not generalize to Medicare populations not seeking services or to other populations. Our study required individuals to have an *ICD-10-CM* OUD diagnosis code; it is possible that some individuals with OUD were not recorded with an OUD diagnosis and thus not included in this study. The provision of telehealth may be underestimated because differential uptake by clinicians and health systems of telehealth billing codes added during the initial phases of the pandemic may have occurred. Given the observational nature of the study, we cannot draw causal inferences.

## Conclusions

This cohort study found that receipt of OUD-related telehealth services was associated with a reduced risk for fatal drug overdose, as was receipt of MOUD from OTPs and receipt of buprenorphine in office-based settings, demonstrating the potential benefits of continuing these services.

## Notes

Supplement 1. **eFigure.** Cohort Pre-Index, Index, and Follow-up Periods for the Pre-Pandemic and Pandemic Cohorts **eTable.** Definitions and Billing Codes Used for OUD, Medications for Opioid Use Disorder, Telehealth, Fatal Drug Overdose, and Other Conditions and Services Received

Supplement 2.
Data Sharing Statement

## References

[1] Public health emergency. Determination that a public health emergency exists. US Department of Health and Human Services. January 31, 2020. Accessed February 17, 2023. https://www.phe.gov/emergency/news/healthactions/phe/Pages/2019-nCoV.aspx

[2] COVID-19 disaster declarations. Federal Emergency Management Agency (FEMA). Accessed February 17, 2023. https://www.fema.gov/disaster/coronavirus/disaster-declarations

[3] Medicare payment policies during COVID-19. Centers for Medicare and Medicaid Services. Accessed February 17, 2023. https://telehealth.hhs.gov/providers/billing-and-reimbursement/medicare-payment-policies-during-covid-19/

[4] Chu RC, Peters C, De Lew N, Sommers BD. Issue brief: state Medicaid telehealth policies before and during the COVID-19 public health emergency. Assistance Secretary for Planning and Evaluation (ASPE). July 2021. Accessed February 17, 2023. https://aspe.hhs.gov/sites/default/files/2021-07/medicaid-telehealth-brief.pdf

[5] Opioid treatment program (OTP) guidance. March 16, 2020. Substance Abuse and Mental Health Services Administration (SAMHSA). Accessed February 17, 2023. https://www.samhsa.gov/sites/default/files/otp-guidance-20200316.pdf

[6] U.S. Department of Justice/Drug Enforcement Administration. Dear Registrant: [This letter only addresses the topic of prescribing buprenorphine for maintenance and detoxification treatment.]. Accessed February 17, 2023. https://www.deadiversion.usdoj.gov/GDP/(DEA-DC-022)(DEA068)%20DEA%20SAMHSA%20buprenorphine%20telemedicine%20%20(Final)%20+Esign.pdf

[7] Jones CM, Shoff C, Hodges K, . Receipt of telehealth services, receipt and retention of medications for opioid use disorder, and medically treated overdose among beneficiaries before and during the COVID-19 pandemic. JAMA Psychiatry. 2022;79(10):981-992. doi:10.1001/jamapsychiatry.2022.2284 36044198 PMC9434479

[8] Master Beneficiary Summary File (MBSF) Base. Centers for Medicare & Medicaid Services. Research Data Assistance Center. Accessed February 17, 2023. https://resdac.org/cms-data/files/mbsf-base

[9] Master Beneficiary Summary File (MBSF): 27 CCW Chronic Conditions Segment. Centers for Medicare & Medicaid Services. Research Data Assistance Center. Accessed February 17, 2023. https://resdac.org/cms-data/files/mbsf-27-cc

[10] Williams AR, Nunes EV, Bisaga A, Levin FR, Olfson M. Development of a Cascade of Care for responding to the opioid epidemic. Am J Drug Alcohol Abuse. 2019;45(1):1-10. doi:10.1080/00952990.2018.1546862 30675818 PMC6404749

[11] National Academies of Sciences, Engineering, and Medicine. Medications for Opioid Use Disorder Save Lives. National Academies Press; 2019. 30896911

[12] Medications for Opioid Use Disorder: Treatment Improvement Protocol (TIP) 63. Substance Abuse and Mental Health Services Administration (SAMHSA). Updated 2021. Accessed February 17, 2023. https://store.samhsa.gov/sites/default/files/pep21-02-01-002.pdf

[13] Hedegaard H, Miniño AM, Spencer MR, Warner M. Drug overdose deaths in the United States, 1999–2020. NCHS Data Brief. No. 428. National Center for Health Statistics; 2021.

[14] CMS Behavioral Health Strategy. Centers for Medicare and Medicaid Services (CMS). Last modified May 5, 2022. Accessed February 17, 2023. https://www.cms.gov/cms-behavioral-health-strategy

[15] Overdose Prevention Strategy. US Department of Health and Human Services. Accessed February 17, 2023. https://www.hhs.gov/overdose-prevention/

