# Supplementary material for: Association of Receipt of Opioid Use Disorder–Related Telehealth Services and Medications for Opioid Use Disorder With Fatal Drug Overdoses Among Medicare Beneficiaries Before and During the COVID-19 Pandemic
Source: JAMA Psychiatry. 2023 Mar 29;80(5):508–14. doi: 10.1001/jamapsychiatry.2023.0310 (PMC10061313; doi:10.1001/jamapsychiatry.2023.0310)
Supplement: Supplement 1. — eFigure. Cohort Pre-Index, Index, and Follow-up Periods for the Pre-Pandemic and Pandemic Cohorts eTable. Definitions and Billing Codes Used for OUD, Medications for Opioid Use Disorder, Telehealth, Fatal Drug Overdose, and Other Conditions and Services Received [file jamapsychiatry-e230310-s001.pdf]

Supplemental Online Content

Jones CM, Shoff C, Blanco C, Losby JL, Ling SM, Compton WM. Association of receipt of opioid use disorder–related telehealth services and medications for opioid use disorder with fatal drug overdoses among Medicare beneficiaries before and during the COVID-19 pandemic. *JAMA Psychiatry*. Published online March 29, 2023. doi:10.1001/jamapsychiatry.2023.0310

eFigure. Cohort Pre-Index, Index, and Follow-up Periods for the Pre-Pandemic and Pandemic Cohorts

eTable. Definitions and Billing Codes Used for OUD, Medications for Opioid Use Disorder, Telehealth, Fatal Drug Overdose, and Other Conditions and Services Received

This supplemental material has been provided by the authors to give readers additional information about their work.

© 2023 Jones CM et al. *JAMA Psychiatry.*

eFigure 1. Cohort Pre-Index, Index, and Follow-up Periods for the Pre-Pandemic and Pandemic Cohorts

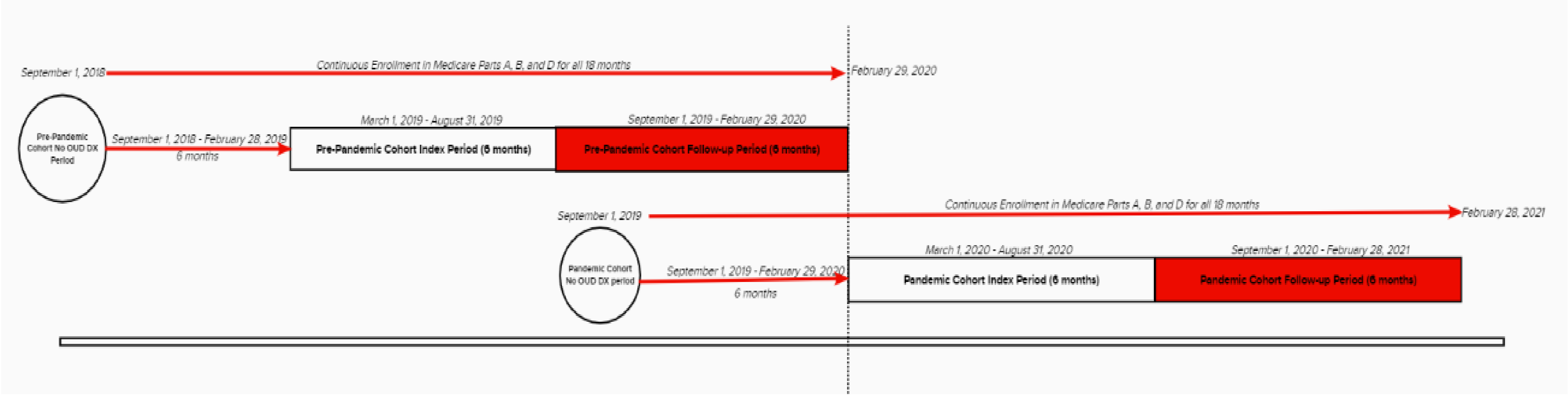

© 2023 Jones CM et al. *JAMA Psychiatry.*

eTable 1. Definitions and Billing Codes Used for OUD and Other Conditions and Services Received

Variables	Definition
**Time Spent in Nursing Home**	We considered an individual to be a current nursing home resident on any given day if the most recent assessment was not a discharge assessment and less than 150 days old. An individual without an assessment for 150 days was assumed to be discharged from the facility.
**Medication Received**	**NDCs**
Buprenorphine in office-based settings	59385001201, 59385001230, 59385001401, 59385001430, 59385001601, 59385001630, 00093365640, 00093365740, 00093365840, 00093365940, 42858035340, 42858049340, 42858050103, 42858050203, 42858058640, 42858075040, 42858083940, 55700057904, 58118315608, 60687048121, 60687049221, 62756045964, 62756045983, 62756046064, 62756046083, 67046099430, 67046099530, 70518071100, 70518071101, 70518071102, 70518155700, 70518162500, 70518221700, 70518221800, 71335035301, 71335035302, 71335035303, 71335035304, 71335035305, 71335035306, 71335035307, 71335095001, 71335095002, 71335095003, 71335095004, 71335095005, 71335095006, 71335095007, 71335115401, 71335115402, 71335115403, 71335115404, 71335115405, 71335115406, 71335115407, 71335115408, 71335115409, 55700056804, 00093360040, 00093360140, 00093360240, 00093360340, 67046099230, 67046099330, 60846097003, 60846097103, 63629727001, 63629727002, 67046099830, 67046099930, 00054017613, 00054017713, 00074201201, 00093537856, 00093537956, 00143924605, 00228315303, 00228315603, 00378092393, 00378092493, 00409201232, 21695051510, 35356055530, 35356055630, 40042001001, 42023017901, 42023017905, 43063066706, 50383092493, 50383093093, 54569657800, 55390010010, 55700030230, 55700030330, 58118017608, 58118017708, 63629712601, 63629712602, 63629712603, 63629712604, 63629712605, 63629712606, 63629712607, 63629712608, 63629712609, 68258299103, 68308020230, 68308020830, 70518044200, 70518201400, 70518221600, 70518222600, 71335116301, 71335116302, 71335116303, 71335116304, 71335116305, 71335116306, 71335116307, 71335116308, 71335116309, 67046099030, 67046099130, 64725093003, 64725192403, 63629712501, 63629712502, 63629712503, 63629712504, 63629712505, 63629712506, 63629712507, 69189059101, 67046099630, 67046099730, 70518065200, 70518065201, 70518065202, 00054018813, 00054018913, 00093572056, 00093572156, 00228315403, 00228315473, 00228315503, 00228315573, 00378876793, 00378876893, 00406192303, 00406192403, 00406800503, 00406802003, 00781721664, 00781722764, 00781723864, 00781724964, 42291017430, 42291017530, 43598057930, 43598058030, 43598058130, 43598058230, 47781035503, 47781035603, 47781035703, 50268014415, 50268014515, 50383028793, 50383029493, 52427069203, 52427069403, 52427069803, 52427071203, 53217013830, 54569640800, 55700018430, 60429058630, 60429058730, 62175045232, 62175045832, 62756096964, 62756096983, 62756097064, 62756097083, 65162041503, 65162041509, 65162041603, 65162041609, 70518100700, 70518168400, 70518231100, 70518232700, 71335129601, 71335129602, 71335137801, 58284010014, 52440010014, 12496010001, 12496030001, 00490005100, 00490005130, 00490005160, 00490005190, 12496120201, 12496120203, 12496120401, 12496120403, 12496120801, 12496120803, 12496121201, 12496121203, 12496128302, 12496130602, 16590066605, 16590066630, 16590066705, 16590066730, 16590066790, 23490927003, 23490927006, 23490927009, 35356000407, 35356000430, 43063018407, 43063018430, 49999039507, 49999039515, 49999039530, 52959030430, 52959074930, 54569549600, 54569573900, 54569573901, 54569573902, 54569639900, 54868570700, 54868570701, 54868570702, 54868570703, 54868570704, 54868575000, 55045378403, 55700014730, 55887031204, 55887031215, 63629403401, 63629403402, 63629403403, 63874108403, 63874108503, 66336001530, 66336001630, 68071138003, 68071151003, 68258299903, 12496127802, 12496131002, 49999063830, 49999063930, 63629409201,

	63874117303, 63874117403, 54123011430, 54123090730, 54123091430, 54123092930, 54123095730, 54123098630, 00228315567, 00378876716, 00378876816, 00781721606, 00781722706, 00781723806, 00781724906, 12496010002, 12496010005, 12496030002, 12496030005, 43063075306, 43598057901, 43598058001, 43598058101, 43598058201, 47781035511, 47781035611, 47781035711, 47781035811, 50090292400, 50268014411, 50268014511, 52427069211, 52427069411, 52427069811, 52427071211, 53217024630, 60429058611, 60429058633, 60429058711, 60429058733.
ER naltrexone in office-based settings	NDCs: 00406009203, 43063046915, 52125072702, 63459030042, 63629530402, 65757030001
Methadone from OTPs	HCPCS: G2067, G2078
Buprenorphine from OTPs	HCPCS: G2068, G2069, G2070, G2071, G2072, G2079
ER naltrexone from OTPs	HCPCS: G2073
**OUD-Related Services**	**ICD-10 CM Diagnosis Codes on Part A or B Service Claim**
OUD-Related Services	F1110, F1111, F11120, F11121x, F1114, F1115x, F1118x, F1119, F112x F1122x, F1123, F1124, F1125x, F1128x, F1129
**Telehealth Services**	Only services that are on the Medicare telehealth services list are classified as telehealth eligible and those HCPCS codes billed with the POS = "02" or "95", "GT", "GQ", or "GO" modifiers are considered delivered via a telecommunication system and not in-person. Virtual check-ins were identified using HCPCS codes G2010, G2012 or G0071, as reported by the health care professional on the Part B Claim. E-visits were identified using the CPT codes 99421-99423 or HCPCS codes G2061-G2063, as reported by the rendering health care professional on the Part B Claim.
**Fatal Drug Overdose**	ICD-10 Codes: X40-44, X60-64, X85, Y10-11
**Co-occurring conditions**	**ICD-10 CM Diagnosis Codes**
Substance use disorder	
Alcohol use disorder	F101X, F102X, F109X
Tobacco use disorder	F17
Cannabis use disorder	F121X, F122X, F129X
Cocaine use disorder	F141X, F142X, F149X
Sedative/hypnotic use disorder	F131X, F132X, F139X
Stimulant use disorder	F151X, F152X, F159X
Other psychoactive substance use disorder	F161X, F162X, F169X, F181X, F182X, F189X, F191X, F192X, F199X
Mental health diagnosis	**CMS Chronic Condition Warehouse (CCW) Flags: https://www2.ccwdata.org/web/guest/condition-categories**
Anxiety	Anxiety Disorders CCW Flag
Bipolar disorder	Bipolar Disorder CCW Flag
Major depression	Depression CCW Flag
Personality disorder	Personality Disorders CCW Flag
Attention deficit hyperactivity Disorder	ADHD, Conduct Disorders, and Hyperkinetic Syndrome CCW Flag
Post-traumatic stress disorder	Post-Traumatic Stress Disorder CCW Flag
Schizophrenia or other psychotic disorder	Schizophrenia and Other Psychotic Disorders CCW Flag
Chronic medical conditions	
Cancer	CCW Flags for: Breast Cancer, Colorectal Cancer, Endometrial Cancer, Lung Cancer, Prostate Cancer, Leukemias and Lymphomas
Diabetes	Diabetes CCW Flag
Cardiovascular and other circulatory	CCW Flags for: Atrial Fibrillation, Acute Myocardial Infarction, Ischemic Heart Disease, Heart Failure, Hypertension
Chronic respiratory disease	CCW Flags for: Asthma, Chronic Obstructive Pulmonary Disease, Bronchiectasis

© 2023 Jones CM et al. *JAMA Psychiatry.*

Viral hepatitis	Viral Hepatitis CCW Flag
HIV	HIV/AIDS CCW Flag
Obesity	Obesity CCW Flag
Liver disease, cirrhosis, and other liver conditions, excluding hepatitis	CCW Flags for: Liver Disease, Cirrhosis, and Other Liver Conditions (excluding Hepatitis)
Acute/chronic pain	CCW Flags for: Migraine and Chronic Headache, Fibromyalgia and Chronic Pain and Fatigue, Rheumatoid Arthritis/Osteoarthritis, Multiple Sclerosis and Transverse Myelitis, Sickle Cell Disease, Spinal Cord Injury, Pressure Ulcers and Chronic Ulcers

© 2023 Jones CM et al. *JAMA Psychiatry.*
