# Supplementary material for: Association of Receipt of Opioid Use Disorder–Related Telehealth Services and Medications for Opioid Use Disorder With Fatal Drug Overdoses Among Medicare Beneficiaries Before and During the COVID-19 Pandemic
Source: JAMA Psychiatry. 2023 Mar 29;80(5):508–14. doi: 10.1001/jamapsychiatry.2023.0310 (PMC10061313; doi:10.1001/jamapsychiatry.2023.0310)
Supplement: Supplement 2. — Data Sharing Statement [file jamapsychiatry-e230310-s002.pdf]

Data Sharing Statement

Jones. Association of Receipt of Opioid Use Disorder-Related Telehealth Services and Medications for Opioid Use Disorder With Fatal Drug Overdoses Among Medicare Beneficiaries Before and During the COVID-19 Pandemic. *JAMA Psychiatry*. Published March 29, 2023. doi:10.1001/jamapsychiatry.2023.0310

Data
Data available: No
